# Levamisole-Induced Vasculitis

**Published:** 2018-02-19

**Authors:** Lohrasb Sayadi, Donald Laub

**Affiliations:** University of California, Irvine, Irvine California and University of Vermont, Burlington Vermont

**Keywords:** levamisole, drug-induced vasculitis, Drug abuse, drug complications, gangrene

## DESCRIPTION

**Figure 1:**
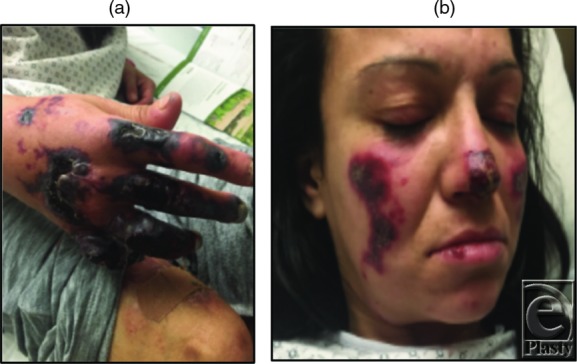
(a) Dry gangrene of the patient's right hand. (b) Gangrene of the patient's cheeks and nasal tip.

T.O. was a 39-year-old woman with a history of intravenous drug abuse, hepatitis C, and hypothyroidism who presented with eschar of her face, lower extremity, and right hand (Figs 1*a* and 1*b*). She indicated that skin lesions began shortly after smoking crack cocaine. Physical examination revealed dry necrotic tissue over the dorsum of her right hand and right ring and small fingers, with the absence of sensation to touch over these regions. Laboratory findings were significant for leukopenia, microcytic anemia, neutropenia, elevated erythrocyte sedimentation rate (ESR), positive lupus anticoagulant, positive anti-nuclear antibody (ANA), and positive antineutrophil cytoplasmic antibodies (p-ANCA). Punch biopsy of her skin demonstrated leukocytoclastic vasculitis with associated intravascular fibrin thrombi, consistent with the diagnosis of levamisole-induced vasculitis (LIV).

## QUESTIONS

1. What is levamisole?
2. What is LIV and what are its clinical findings?
3. What is the mechanism of LIV and what laboratory abnormalities are seen?
4. How can LIV be treated?

## DISCUSSION

Levamisole is a synthetic imidazathiazole antihelminthic agent once used medically for its immunomodulatory effects; its use in the medical setting was curtailed in 1999 due to associated LIV, agranulocytosis, thrombocytopenia, and arthritis.^1^ Other associated effects include multifocal leukoencephalopathy as well as type I and III hypersensitivity reactions.^2^ Although its medical use has been discontinued, it is estimated that 1.5% of the US population uses cocaine regularly and that up to 70% of street cocaine is contaminated with levamisole, making it a significant public health issue.^3^ It is thought that levamisole acts as a cocaine diluent and may also heighten the euphoric effects of cocaine.^4^ The mechanism by which it potentiates the effects of cocaine is due to inhibition of monoamine oxidase and catechol-*O*-methyl transferase, increasing synaptic transmission of norepinephrine and augmenting the sympathomimetic effects of cocaine.^2^ Levamisole is partially metabolized into an amphetamine-like compound and inhibits acetylcholinesterase activity, increasing endogenous opioids and dopamine levels in cerebral reward pathways.^2^

Levamisole is associated with 3 clinical syndromes: cocaine-induced midline destructive lesions, cutaneous vasculitis (LIV), and agranulocytosis.^5^ Levamisole-induced vasculitis is a cutaneous vasculitis that has been reported with smoked crack cocaine and inhaled cocaine powder. It has a greater frequency in women (male to female ratio 1:3), with a mean age of presentation of 44 years.^6^ Although other compounds can be added to cocaine, vasculitis has been associated with long-term use of cocaine mixed with stimulants such as levamisole.^1^ Patients with LIV develop tender purpuric lesions 1 to 3 days after exposure.^4^ These lesions first develop as symmetric erythema, evolve into retiform purpura, bullae, and finally undergo necrosis and eschar formation.^4^ Most commonly, lesions develop over the ear, malar eminences, and tip of the nose. Diagnosis of LIV is on its clinical presentation and histopathological findings of leukocytoclastic vasculitis of small vessels containing fibrinoid necrosis of the vessel wall, erythrocyte extravasation, and multiple fibrin thrombi within small vessels in the superficial and deep dermis.^6^

The exact mechanism by which LIV occurs has not been fully determined. However, LIV is associated with the generation of autoantibodies such as p-ANCA, ANA, and lupus anticoagulant. Levamisole acts as a haptan, triggering an immune response and upregulation of antibody formation. Consequently, it is thought that LIV develops due to immune complex deposition (IgM, IgG, IgA, and C3 complexes) and secondary hypercoagulability caused by the immune complexes, leading to tissue thrombosis and skin necrosis.^3^ A specific genotype of human leukocyte adhesion factor, HLA B27, has been indicated as a risk factor for development of LIV.^2^ Patients who present with agranulocytosis are often ANA positive. In LIV, p-ANCA is more common than c-ANCA.^2^ Proteinase 3 Antibodies (Anti-PR3), Myeloperoxidase Antibodies (Anit-MPO) and Anti-4 Hydroxynonenal antibody (Anti-HNE) distinguish LIV from the other conditions associated with levamisole. Testing for levamisole can be difficult, as its half-life is 5.6 days and it is not part of routine toxicology screening.

Management of LIV is supportive.^4^ The most effective treatment of any of the levamisole-induced syndromes is the cessation of cocaine use.^2^ In addition, steroids may be beneficial.^2^ Other treatments that have been advocated include nonsteroidal anti-inflammatories and colchicine. Surgical debridement of necrotic tissue, amputation, skin grafting, and local wound care are typically necessary for patients with advanced lesions.^6^

**Figure 2:**
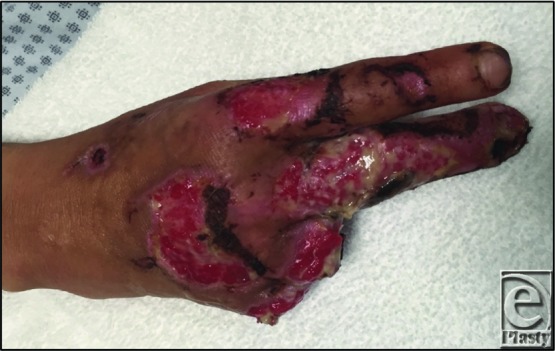
The patient's right hand after debridement and amputation of the fourth and fifth fingers.

Our patient presented with advanced necrotic lesions of her hand, face, and lower extremity. The diagnosis of LIV was supported by reported development of lesions immediately after smoking crack cocaine. Her laboratory values (agranulocytosis, + ANA, + p-ANCA, + c-ANCA, + lupus anticoagulant) and skin biopsy showing leukocytoclastic vasculitis with associated intravascular fibrin thrombi were confirmatory for LIV. She underwent debridement and amputations of 2 fingers (Fig 2) and later skin grafting. She was counseled against future substance abuse and elected to receive outpatient drug rehabilitation.
